# The emerging role of long non-coding RNAs in renal cell carcinoma progression and clinical therapy *via* targeting metabolic regulation

**DOI:** 10.3389/fphar.2023.1122065

**Published:** 2023-03-09

**Authors:** Xingyu Gao, Haiying Zhang, Chang Zhang, Minghe Li, Xiao Yu, Yanan Sun, Yingai Shi, Hongxia Zhang, Xu He

**Affiliations:** ^1^ The Key Laboratory of Pathobiology, Ministry of Education, College of Basic Medical Sciences, Jilin University, Changchun, China; ^2^ Reproductive Medicine Center, China-Japan Union Hospital, Jilin University, Changchun, China; ^3^ Department of Rehabilitation Medicine, China-Japan Union Hospital, Jilin University, Changchun, China

**Keywords:** renal cell carcinoma, long non-coding RNAs, metabolism, biomarkers, tumor progression

## Abstract

Renal cell carcinoma (RCC) is the most frequent renal malignancy in the world, and its incidence is increasing year by year. RCC is a well-known drug resistant tumor, and the treatment methods are limited. Most patients with RCC are discovered at the advanced stage, and thus have poor prognosis even after treatment. Therefore, it is very urgent to find new markers for the diagnosis and treatment of RCC. Accumulating evidence shows that lncRNAs participate in the occurrence and progression of RCC, which is achieved by the lncRNA-miRNA-mRNA axis. It is widely known that metabolic defect is an essential pathogenesis in RCC. As is the case with other tumors, RCC can satisfy the demands of cancerous cells for uncontrolled proliferation through aerobic glycolysis. However, whether lncRNAs can modulate RCC progression through metabolic pathway is still not clarified. Taken together, this review mainly summarized the metabolic regulatory mechanisms of lncRNAs in RCC progression, especially their roles in glucose metabolism, lipid metabolism, amino acid metabolism and mitochondrial dynamics, as well as the clinical applications of lncRNAs *via* targeting metabolism in RCC therapy. It will provide the new targets and approaches for early clinical diagnosis, treatment and prognosis of RCC.

## Graphical Abstract

**Figure:**
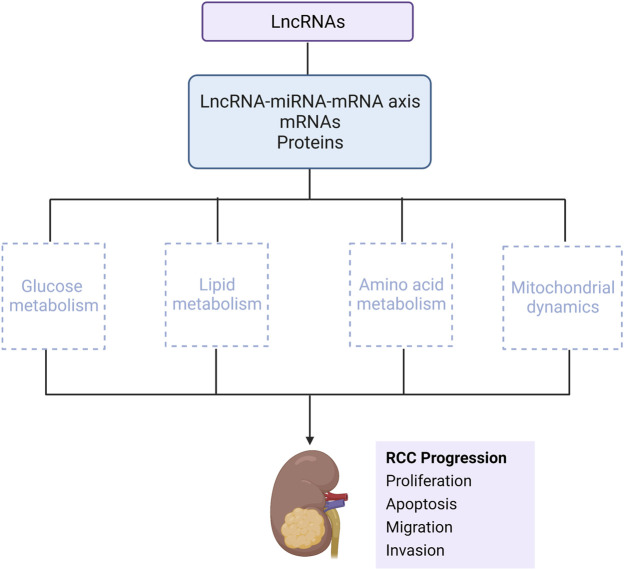
This review mainly describes that lncRNAs interact with miRNA-mRNA axis, or directly binds to mRNAs and proteins, to influence RCC progression *via* metabolic regulation, mainly including glucose metabolism, lipid metabolism, amino acid metabolism and mitochondrial dynamics (Created with biorender.com).

## 1 Introduction

Renal cell carcinoma (RCC) is currently the 12th most frequent cancer in the world, accounting for 3% of adult cancers, 2% of deaths (Iaxx et al., 2022), and 2.4% of all cancer diagnoses worldwide (Makino et al., 2022). Renal parenchymal malignant tumors originate from renal tubular epithelial cells. In 2016, WHO classified renal carcinomas based on their morphological, molecular and genetic characteristics. The most common subtype is clear cell renal carcinoma (ccRCC), and other common subtypes include papillary RCC (further divided into types 1 and 2, pRCC), chromophobe RCC (chRCC) with favorable outcome, and the collecting duct carcinoma with the most adverse prognosis (Ganini et al., 2022; Gulati et al., 2022). As a malignant tumor, RCC has strong immunogenicity, which is infiltrated by a large number of T cells, B cells, macrophages and other immune cells. Because RCC is insensitive to traditional radiotherapy and chemotherapy, immunotherapy has leaped to the forefront of treatment in recent years (Cao et al., 2022). However, due to tumor heterogeneity and drug resistance, immunotherapy lacks a complete anti-tumor response. In the course of clinical treatment, about 30% of RCC patients have metastasis at the time of diagnosis, and 30%–70% of RCC patients are still likely to relapse after surgical treatment (Xiang et al., 2022). As a result, it is essential to better understand RCC pathogenesis and progression-related mechanisms to search for the new strategies for the treatment of this fatal malignancy.

Long non-coding RNA (LncRNA) is a transcript encoded by eukaryotic cell genome, with a length more than 200 nucleotides, which is crucial for cell proliferation, tumorigenesis and inflammatory response (Yang et al., 2021). In most cases, the expression of lncRNAs is generally lower than that of protein encoded RNAs in human tissues. Unlike protein encoded genes, lncRNAs do not contain functional open reading frames and have short conserved sequences to maintain their functional and structural stability (Tang et al., 2017). Although lncRNAs lack the ability of protein coding, they are still transcribed by RNA polymerase II, and then undergo posttranscription capping, splicing and polyadenylation (Shen et al., 2021). The main functions of lncRNAs include X chromosome silencing, transcriptional activation, transcriptional interference, tumor regulation, and maintenance of RNA stability (Yao et al., 2022). More and more evidence shows that highly structured lncRNAs bind to chromatin regulators and change their state, and lncRNAs participate in the occurrence of tumor and the expression disorder of protein coding genes in tumors by interacting with transcription factors, RNAs and miRNAs (Rinn and Chang, 2012). These processes will directly affect the level of messenger RNA (mRNA) synthesis of protein coding genes, alternative splicing process, stability of mRNAs, translation level and protein stability (Braga et al., 2021). X-Inactive-Specific-Transcript (XIST) is one of the first discovered lncRNAs by human beings, which is considered to be the key gene involved in the process of X chromosome inactivation in mammals or the only one affected by X chromosome inactivation. X chromosome inactivation is a unique developmental regulatory mechanism, mainly through the random inactivation of the female X chromosome, to maintain the balance between male and female X chromosomes (Brown et al., 1991). The roles of lncRNAs in RCC have been extensively studied, including the regulation of apoptosis, cell cycle, cell proliferation, invasion and migration in the form of oncogenes or tumor suppressor genes (Li et al., 2017). LncRNAs have been considered as one of the cancer biomarkers, closely related to the occurrence and development of tumors, which are of great significance for tumor diagnosis, treatment and prognosis.

Metabolism is the most basic process in life activity of organisms. Normal cells metabolize through oxidative phosphorylation under aerobic conditions, while tumor cells obtain energy mainly through glycolysis (Huang et al., 2022). Abnormal cell metabolism is an important hallmark of tumorigenesis and progression. A large number of studies have demonstrated that metabolism in ccRCC is different from other subtypes of renal carcinoma. Because the cytoplasm of ccRCC contains numerous lipids and glycogen, the cell body appears translucent, which also confirms why ccRCC is a metabolic disordered disease (Bacigalupa and Rathmell, 2020). Meanwhile, metabolic diseases such as type 2 diabetes, hypertension, obesity and lipid disorders can enhance the risk of RCC (Gluba-Brzózka et al., 2022; Graff et al., 2022). The above results indicate that metabolic abnormalities exist in RCC, which may be one of the main factors affecting RCC progression. However, the underlying mechanisms that lncRNA influence RCC progression through metabolism regulate remain unclear.

## 2 The role of lncRNAs in RCC

In the diagnosis of cancers, lncRNAs have been identified as active biomarkers, which play a carcinogenic role by regulating the biological characteristics of cancer, such as cell proliferation, migration, invasion and apoptosis. The pathogenesis of lncRNAs in RCC are extensively studied. As the most studied oncogene, lncRNAs act on the genes through the lncRNA-miRNA-mRNA axis. In addition, lncRNAs also bind directly to proteins and genes/DNA. Hence, it is imperative to clarify how lncRNAs can be used as new markers and therapeutic targets for RCC.

### 2.1 LncRNAs are up-regulated in RCC

A TCGA analysis of clinical samples showed that LINC00406 was up-regulated in RCC, and LINC00406 over-expression could enhance cell proliferation, migration and invasion, and inhibit RCC apoptosis. It was further confirmed that PI3K/AKT signal pathway, as the downstream of LINC00406, affected RCC progression (Zhou et al., 2022). The in-depth regulatory mechanism still needs to be further explored. DLEU7-AS1 is considered to be a carcinogene in colorectal cancer. However, the latest research found that DLEU7-AS1 expression was significantly elevated in RCC. Double luciferase reporter assay further confirmed that miR-26a-5p was the direct target of DLEU7-AS1 (Wang X. J. et al., 2022). The promotion effect of lncRNA FTX on the malignant phenotypes of RCC has also been testified. FTX significantly facilitated the cell viability, proliferation, migration and invasion of RCC, and exerted a carcinogenic role through FTX-mir4429-UBE2C axis. FTX is expected to become a promising therapeutic target for RCC (Chen et al., 2022b). Wang et al. found that lncRNA CYTOR (cytoskeleton regulator RNA) was highly expressed in 786-O and Caki-1 cell lines, which was negatively related to the prognosis of RCC. The interaction between CYTOR and miR-136-5p was further verified by double luciferase reporter and RNA pull down assays (Wang D. et al., 2022). Another study reported that lncRNA MMP2-AS1 was highly abundant in tumor samples of RCC patients. It was then demonstrated that knockdown of MMP2-AS1 could inhibit the proliferation of RCC *in vitro* and *in vivo*. Furthermore, miR-34c-5p inhibitor reversed the inhibition of MMP2-AS1 depletion on RCC (Fan et al., 2022).

Tumor derived exosomes can regulate cancer cell progression and macrophage polarization. Numerous studies have found that RCC derived exosomes promote macrophage polarization, angiogenesis and tumor progression. More importantly, exosomes from RCC contain a large amount of lncRNA ASAR, which lead to the transformation of macrophages from M1 to M2. M2 macrophages can release a large number of anti-inflammatory factors, and activates the miR34/miR449/STAT3 pathway, thus providing an appropriate microenvironment for tumor metastasis (Zhang W. et al., 2022). In other studies, patients with advanced RCC have been found to be resistant to sunitinib, so it is indispensable to explore new targets of sunitinib resistance. ASAR had been confirmed to be highly expressed in advanced RCC, and promoted sunitinib resistance through competitive binding with miR34/miR449 (Qu et al., 2016). In addition, lncRNA SNHG12 could not only accelerate RCC progression, but also facilitate sunitinib resistance. SNHG12 competitively bound with miR-129-5p to regulate MAPK/ERK signaling pathway and G1/S cell cycle transition (Feng et al., 2022). SNHG12 also contributed to sunitinib resistance by reducing SP1 ubiquitination mediated proteolysis and up-regulating CDCA3 expression through binding to transcription factor SP1 (Liu et al., 2020). Another study revealed that SNHG12 interacted with miR-30a-3p and up-regulated the expression of several target genes, including RUNX2, WNT2 and IGF-1R. Both *in vivo* and *in vitro* experiments confirmed that SNHG12 induced ccRCC progression by a crosstalk relationship with target genes (Yu et al., 2021). So far, the research conclusion on SNHG12 is relatively consistent. SNHG12 knockout in A498 and 786-O cell lines attenuated RCC cell viability and promoted cell apoptosis, which is achieved by sponging miR-200c-5p (Xu et al., 2020). The treatment options for patients with advanced metastatic RCC are limited. The main common drugs are Sunitinib and sorafenib are commonly used drugs, but drug resistance usually occurs after 5–6 months of administration. Therefore, it is very urgent to explore the mechanisms of sunitinib resistance and its biomarkers. SNHG12 is a newly discovered biomarker of sunitinib resistance in recent years, and its underlying mechanism still needs to be further investigated, so as to provide a theoretical basis for clinical diagnosis, treatment and prognosis of RCC.

### 2.2 LncRNAs are down-regulated in RCC

The biological function of lncRNA XIST (X-inactive specific script) is currently controversial in the RCC progression. It was reported that, compared with normal tissues, XIST was significantly down-regulated in RCC cell lines (ACHN, CAKI-1, CAKI-2 and 786-O). Over-expression of XIST *in vitro* experiments remarkably inhibited cell viability and induced cell cycle to stagnate at G0/G1 phase. *In vivo* experiments manifested that the size and weight of the tumors were remarkably reduced when the ACHN cells transfected with XIST were injected into mice. The study further indicated that curcumin, a plant component with antineoplastic properties, played an anti-tumor role in RCC by activating the expression of XIST and regulating XIST/miR-106b-5p/p21 axis (Sun et al., 2019). However, another study demonstrated that XIST was conspicuously up-regulated in RCC cell line 786-O compared with normal renal tubular epithelia, and the same results were obtained in RCC cell lines CAKI-1, ACHN and 769-P. Knockdown of XIST obviously attenuated the proliferation, migration and invasion of RCC, and facilitated cell apoptosis. XIST regulated RCC progression through miR302c/Syndecan-1 (SDC1) axis (Zhang et al., 2017). So far, the regulatory effects of XIST on RCC progression have not been extensively studied. In recent years, there are only these two papers, but their results are completely opposite. More studies on the relationship between XIST and RCC progression are expected, and the underlying mechanisms will be gradually unraveled.

There was evidence that N6 methyl adenosine (m6A) modification mediated tumor progression of RCC by regulating lncRNA NEAT1. M6A modification is a common mRNA modification that regulates RNA translation, location and stability. As a key member of m6A methyltransferase complex, the expression of Methyltransferase like 14 (METTL14) was down-regulated in RCC (Liu T. et al., 2022). At the same time, NEAT1 expression and m6A methylation level in RCC tissue decreased compared with normal kidney. When NEAT1 was methylated by CRIPSR/dCas13b-METTL3, NEAT expression was up-regulated and RCC proliferation was suppressed (Chen J. et al., 2021). Nevertheless, another study revealed that NEAT1 expression was significantly higher in RCC cell lines than that in non-tumor cells, with the highest expression in 786-O and ACHN cell lines. NEAT1 knockdown enhanced the expression of miR-34a, indicating that NEAT1 may play a role as the ceRNA of miR-34a (Liu et al., 2017).

The expression of lncRNA KCNQ1DN was confirmed to be down-regulated in RCC tissues and cell lines. Methylation analysis showed that hypermethylation of the KCNQ1DN promoter site might be responsible for partial or complete KCNQ1DN silencing in RCC. Concurrently, KCNQ1DN restrained RCC progression by regulating the transcription factor c-MYC and downstream targets cyclin D1 and p27 (Yang et al., 2019). However, the view that regional hypermethylation will lead to the activation of proto-oncogenes and abnormal gene expression has also been verified in many other tumor suppressors (Shenoy et al., 2015). Another tumor suppressor, lncRNA-NR_ 023387 possessed an inhibitory effect on RCC proliferation. In RCC, due to the hypermethylation of CpG island and the lack of hepatic nuclear factor 4 α (HNF4A), NR_ 023387 expression was silenced. Matrix Gla protein (MGP) is the downstream molecule of its carcinogenesis (Zhou et al., 2020). High expression of DNA methyltransferase 3A (DNMT3A) at the lncRNA SLERCC (Specific Low Expression in RCC) promoter site will result in abnormal hypermethylation, which ultimately leads to decreased SLERCC expression in RCC. SLERCC inhibits RCC progression and metastasis through SLERCC-Up-frameshift protein 1 (UPF1)—Wnt/β—catenin axis (Mao et al., 2022) (Table 1).

**TABLE 1:** The expression of lncRNAs and regulatory mechanisms in RCC.

LncRNA	Expression of RCC	Mechanisms	Biological behavior of RCC	References
LINC00406	up-regulated	PI3K\AKT	proliferation	Zhou et al. (2022)
LncRNA DLEU7-AS1	up-regulated	miR-26a-5p	proliferation	Wang X. J. et al. (2022)
LncRNA FTX	up-regulated	mir4429-UBE2C	proliferation	Chen et al. (2022b)
LncRNA CYTOR	up-regulated	miR-136-5p	proliferation	Wang D. et al. (2022)
LncRNA MMP2-AS	up-regulated	miR-34c-5p\MMP2	proliferation	Fan et al. (2022)
LncRNA ASAR	up-regulated	miR34\miR449\STAT3	proliferation	Zhang W. et al. (2022)
		miR34\miR449		Qu et al. (2016)
LncRNA SNHG12	up-regulated	miR-129-5p\MAPK\ERK	proliferation	Feng et al. (2022)
		SP1\ CDCA3		Liu et al. (2020)
		miR-30a-3p		Yu et al. (2021)
		miR-200c-5p		Xu et al. (2020)
LncRNA XIST	up-regulated	miR302c\ SDC1	proliferation	Zhang et al. (2017)
	down-regulated	miR-106b-5p/p21	apoptosis	Sun et al. (2019)
LncRNA NEAT1	down-regulated	METTL14	apoptosis	Chen J. et al. (2021), Liu T. et al. (2022)
	up-regulated	miR-34a\c-Met	proliferation	Liu et al. (2017)
LncRNA KCNQ1DN	down-regulated	c-MYC\ cyclin D1\p27	apoptosis	Yang et al. (2019)
lncRNA NR_023387	down-regulated	MGP	apoptosis	Zhou et al. (2020)
LncRNA SLERCC	down-regulated	UPF1-Wnt/β-catenin	apoptosis	Mao et al. (2022)

## 3 The role of metabolism in RCC

Among all cancers, RCC is one of the most ideal models for metabolic reprogramming. In the occurrence and development of RCC, the genes regulating tumor progression participate in the modulation of metabolic processes, such as glycolysis, pentose phosphate pathway (PPP), TCA cycle and ATP generation.

### 3.1 Glucose metabolism

Glucose metabolism principally includes glycolysis, PPP and mitochondrial TCA cycle in the cytoplasm. In normal cells, glucose is transported to cells through transporters, converted into pyruvic acid through glycolysis, and then transmitted to mitochondria to enter the TCA cycle to generate ATP *via* oxidative phosphorylation (OXPHOS), which is the main energy source in biological systems (Triplitt, 2012).

In 1927, Otto Warburg observed metabolic reprogramming in cancer cells for the first time. It was found that the incidence of aerobic glycolysis in cancer cells was higher than that of oxidative phosphorylation. Thus, the respiratory level of oxidative phosphorylation pathway would be inhibited and damaged, which was the characteristic phenomenon of abnormal metabolism in cancer, known as Warburg effect (Hay, 2016). Cancer cells produce lactic acid through aerobic glycolysis to replace the TCA cycle in normal cells (Qi et al., 2021). However, compared with the oxidative phosphorylation pathway, the conversion of glucose to lactic acid produces less energy. It is insufficient to maintain the energy supply for cancer cells, and then more glucose will be consumed (Chakraborty et al., 2021; Jones and Bodenstine, 2022). The kidneys contain a large number of glucose transporters, which participate in glucose reabsorption, excretion and other metabolic pathways. Therefore, the kidneys are essential to maintain glucose homeostasis (Courtney et al., 2018). Proteomic analysis showed that the expression of TCA circulating enzyme in RCC was lower than that of the other tumor types. The mechanism was mainly due to TGF- β combined with histone deacetylase 7 (HDAC7) inhibiting the expression of TCA circulating enzyme. *In situ* models of RCC, TGF-β suppression through pharmacological treatment can up-regulate the expression of TCA circulating enzyme and restrain the proliferation of RCC(Nam et al., 2021). It is worth noting that there is a unique nutrient allocation in the tumor models. Bone marrow cells have the strongest ability to consume glucose in patients with cancer, followed by T cells and cancer cells, while cancer cells have the greatest glutamine uptake capacity. This research puts forward a view contrary to Warburg effect. Glutamine is primarily supplied to tumor cells, while glucose will be firstly provided to immune infiltrative cells around cancer cells (Reinfeld et al., 2021). Nevertheless, up to now, no similar studies have been confirmed, which does not mean that the Warburg effect will be overturned.

**FIGURE 1:**
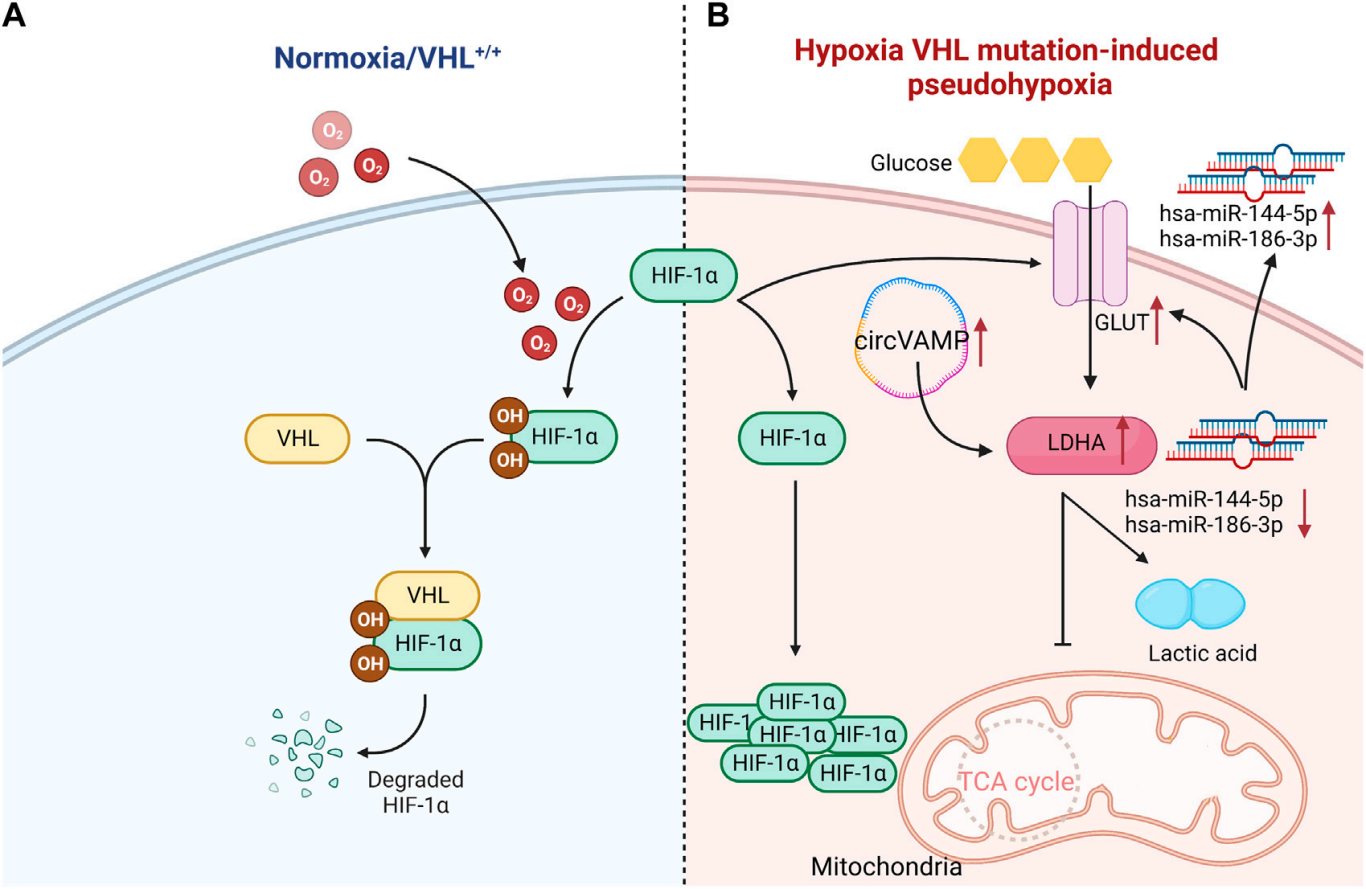
VHL normally codes HIF-1α protein, and targets HIF-1α transcription factors for proteosome degradation under aerobic conditions. Once VHL is lost, the degradation process of transcription factors will be weakened, leading to the accumulation of HIF-1α transcription factors under normal oxygen conditions. Compared with normal renal tubular cells, the mutation mediated by VHL- HIF-1α can up-regulate the expression of GLUT-1 in ccRCC cells. Hsa-miR-144-5p and hsa-miR-186-3p directly bind to 3'UTR of GLUT-1 mRNA, and thus GLUT-1 expression in ccRCC is significantly increased. Meanwhile, circVAMP is highly expressed in ccRCC, which combines with LDHA to inhibit TCA cycle, and then promotes glycolysis to produce lactic acid. Since the conversion of glucose into lactic acid produces less energy than oxidative phosphorylation, it is insufficient to maintain the energy supply for cancer cells. Hence, more glucose will be consumed. ↑:increase; ↓:reduce;˗:inhibition (Created with biorender.com).

Notably, the genes controlling metabolism in ccRCC have a high mutation rate. Clinical studies found that about 90% of patients have the deletion of von Hipple Lindau (VHL) gene in the hypoxia pathway. VHL normally codes E3 ubiquitin ligase that recognizes hypoxia inducible factor-1α (HIF-1α) protein, and VHL targets HIF-1α transcription factors for proteosome degradation under aerobic conditions. Once VHL is lost, the degradation process of transcription factors will be weakened. And then HIF-1α transcription factors under normoxic conditions will be accumulated, and makes cells in a pseudohypoxic state, leading to the changes in the metabolic process (Appelhoff et al., 2004; Chatzopoulos et al., 2022; Chen et al., 2022a; Jones and Bodenstine, 2022). The mutation mediated by VHL/HIF-1α can up-regulate the expression of glucose transporter 1 (GLUT-1) in ccRCC, and the glycolysis related gene lactate dehydrogenase A (LDHA) also strongly expresses in several tumor cell lines and RCC. Unlike other phenotypes of RCC, the infiltration of CD8^+^T cells in ccRCC was negatively correlated with the expression of GLUT-1, which indicated that glycolytic genes played the critical roles in regulating immune cell infiltration in tumor microenvironment (Singer et al., 2011). The expression of GLUT-1 can be manipulated by microRNAs. The expressions of hsa-miR-144-5p and hsa-miR-186-3p in ccRCC were significantly decreased, and both microRNAs could directly bind to GLUT-1 mRNA 3'UTR. Non-etheless, their extracellular expressions were relatively increased. Hence, microRNAs might contribute to the Warburg effect of RCC by enhancing extracellular secretion to reduce the intracellular level (Morais et al., 2021). It was found that circVAMP was the first circRNA discovered to directly interact with LDHA and regulate its activity. CircVAMP was highly expressed in RCC and combined with LDHA to promote glycolysis, thus accelerating the progression of RCC (Li et al., 2022) (Figure 1). Previous studies found that the high expression of LDHA in RCC was also related to the mitochondrial DNA copy number (mtDNA). And the copy number of mtDNA in RCC was significantly reduced, which was proportional to mitochondrial ATP production. This weakened mitochondrial respiration was compensated by the increased glycolysis, thereby affecting the invasive ability of RCC (Lin et al., 2016). A large amount of evidence confirmed that LDHA, as an oncogene, is crucial to the occurrence and progression of RCC. LDHA mainly catalyzes the reduction of pyruvate to lactic acid, which is essential to drive Warburg effect. Consequently, it is of great significance to apply LDHA inhibitors in the clinical treatment of RCC.

### 3.2 Lipid metabolism

The data in 2018 demonstrated that more than 170,000 people died from RCC. The abnormality of lipid metabolism is the major characteristic in ccRCC. In a study, proteomic analysis of tumor tissues at four different stages of ccRCC was carried out. The results displayed that mitochondrial enoyl coenzyme A hydratase (ECHS1) presented significant down-regulation at four stages of ccRCC, which facilitated the proliferation and metastasis of RCC by activating m-TOR signaling pathway in RCC (Wang et al., 2020). Consequently, ECHS1 is expected to become a new target and diagnostic marker for the ccRCC treatment. Sterol regulatory element binding protein (SREBP) is a transcription factor that modulates cholesterol and fatty acid biosynthesis. Wang et al. reported that programmed death receptor ligand 1 (PD-1) over-expression accelerated epithelial mesenchymal transformation (EMT) and the formation of epithelial-derived carcinomas. The main mechanism was to induce EMT through up-regulating SREBP-1c, and ultimately enhance the carcinogenesis of RCC (Wang et al., 2015). Another study indicated that there was a strong correlation between the expression of two lipid metabolism enzymes, hydroxyl-coenzyme A dehydrogenase, alpha subunit (HADHA) and acetyl-coenzyme A acetyltransferase 2 (ACAT2), whose down-regulation gave rise to the poor prognosis in RCC patients (Zhao et al., 2016). The formation of lipid droplets (LD) in tumor cells is the hallmark of RCC, which produces ATP through fatty acid (FA) oxidation. LD not only provides FA in the presence of nutrient and oxygen deprivation, but also maintains cellular redox homeostasis, and prevents cancer cells from ROS destruction by sustaining the NADPH formation (Petan et al., 2018). The main component of lipids is FA. The core steps of long chain FA *de novo* synthesis include *de novo* synthesis and desaturation. Elongase (ELOVL family) and desaturase 1 (SCD1) are vital determinants for FA unsaturated length and degree (Guillou et al., 2010).

Another characteristic of RCC is the accumulation of polyunsaturated fatty acids (PUFA). ELOVL mediates the extension of PUFA and stimulates the invasion and metastasis of RCC. Knockout of ELOVL suppressed AKT Ser473 phosphorylation and the proliferation of RCC through AKT-mTOR-STAT3 signal pathway (Reinfeld et al., 2021). In addition to ccRCC, there is a high level of ELOVL in pRCC and chRCC, which is closely related to the poor prognosis of pRCC patients. Declined expression of ELOVL accelerated the formation of LD in RCC and suppressed the proliferation of cancer cells by affecting lipid metabolism (Tanaka et al., 2022). Liquid chromatography and electrospray ionization-tandem mass spectrometry analysis showed that knockout of ELOVL5 reduced its end products (arachidonic acid and eicosapentaenoic acid), and restrain RCC proliferation *in vitro* by inducing apoptosis. It implied that the elevated ELOVL5 expression would have a negative impact on the clinical prognosis of RCC patients (Nitta et al., 2022). Another lipid metabolism related gene, SCD1, is considered to be crucial to the activity of ccRCC. High levels of SCD1 can promote the proliferation and survival of RCC. In the other tumors such as breast cancer and lung cancer, SCD1 has also been confirmed to be a marker of carcinogenesis (Melana et al., 2021; Yang et al., 2022). SCD1 function is to desaturate saturated fatty acids (SFAs) and involved in the synthesis of monounsaturated fatty acids (MUFA). Increased SCD1 activity destroyed the metabolic balance of SFAs and MUFA, and further facilitated more lipid production (Raeisi et al., 2022). Tumor recurrence is not only the major clinical problem, but also the most common problem in tumor therapy. The clinical trial data verified that SCD1 was significantly up-regulated in the samples of human recurrent breast cancer. SCD1 can protect tumor cells from oxidative stress damage, prevent iron death and contribute to tumor cells regeneration. It was showed that lipid desaturation in tumors played an essential role in drug resistance (Tesfay et al., 2019; Luis et al., 2021; Xuan et al., 2022). One study found that the occurrence of iron death was mostly caused by the preoxidation of PUFA, while SCD1 catalyzed MUFA could repress iron death by replacing PUFA in lipid membrane and reducing ROS accumulation. Additionally, lactic acid could enhance iron death resistance of tumor cells by activating AMPK-sterol regulatory element binding protein 1 (SREBP1) -SCD1 pathway (Zhao et al., 2020). Importantly, because SCD1 was over-expressed in ccRCC, SCD1 inhibition *via* pharmacological and genetic manipulation could lead to the reduction in cell viability due to a diminished proliferation rate (Melana et al., 2021). Furthermore, clinical data manifest that SCD1 can be used as a target gene to regulate the adipogenesis. Hence, it is concluded that blocking SCD1 can also suppress the adipogenic differentiation, which provides a key target for the treatment of metabolic diseases (Zhang et al., 2021).

A large number of studies have confirmed that SCD1 is an oncogene, but there is no evidence demonstrating that SCD1 inhibitors in clinical application can ameliorate patient survival (Liu Y. et al., 2022; Hu et al., 2022; Wang L. et al., 2022). Numerous studies have shown that in clinical applications, SCD1 inhibitors (thiazol-4-acetic acid derivatives) have therapeutic potential for obesity, cancer and diabetes, but it may produce associated clinical adverse reactions (Iida et al., 2018). T-3764518, as a new oral SCD1 inhibitor, can alter lipid metabolism by disrupting the balance of saturated and unsaturated fatty acid in membrane lipid, thus playing an anti-tumor role *in vivo* and *in vitro* (Nishizawa et al., 2017). Nevertheless, a opposite view was proposed that patients with high levels of SCD1 expression were positively correlated with survival. The reason may be that SCD1 inhibitors not only attenuate the growth of cancer cells, but also damage normal cells and tissues, that is, the off-target effect of SCD1 inhibitors (McCauley et al., 2020).

**FIGURE 2:**
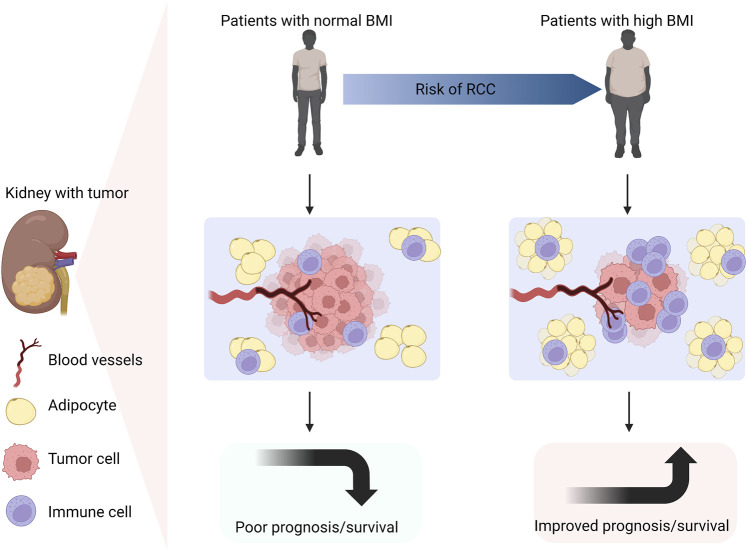
Obesity is an independent risk factor for RCC. Compared with patients with normal BMI, obese patients are more likely to suffer from RCC. Due to the lower invasiveness, higher immunogenicity and more infiltrative inflammatory cells in obese individuals with RCC, a stronger inflammatory reaction will occur. Thus, obese patients with RCC have better prognosis and longer survival (Created with biorender.com).

Excessive calorie intake can result in abnormal accumulation of adipose tissue and triglycerides, causing cell hypertrophy and invading other parts of the body (Wilson and Cho, 2016; Huang et al., 2020). Cancer cells depend on the surrounding non-malignant tumor stromal cells to survive. Adipose tissue exists in cancer cells, which provides necessary energy for cancer cells. Hence, excessive energy and nutrition intake in any way can lead to the accumulation of adipose tissue, which in turn contributes to the malignant proliferation of cancer cells (Darbas et al., 2020; Gluba-Brzózka et al., 2022). The World Cancer Foundation (WCRF) suggests that reasonable diet and proper physical exercise are the best ways to prevent cancer (Kerschbaum and Nüssler, 2019). In addition, there is an interesting phenomenon between RCC and obesity, which is called the “obesity paradox”. Obesity is an independent risk factor for RCC. Whereas, weight loss in obese patients, rather than low body mass index (BMI), is inversely proportional to the mortality of RCC. This result indicates that obesity may benefit the prognosis of RCC patients. The reason is that renal tumors have less invasiveness and higher immunogenicity, which can stimulate stronger inflammatory reaction, and thus provide better therapeutic effect in obese individuals (Sanchez et al., 2020; Graff et al., 2022). It is speculated that this phenomenon is related to abnormal lipid accumulation and metabolism in ccRCC. In 1999, the obesity paradox was first described in overweight and hemodialysis patients. Later, it was explained that obese patients were easier to be diagnosed than normal weight patients, so they would receive the earlier treatment and have the better prognosis (Fleischmann et al., 1999) (Figure 2). Abundant adipocytes could produce a large number of tumor necrosis factor receptor (TNF-α), which neutralized the cardiotoxicity of TNF-α, thus counteracting the negative effects of renal dysfunction (Turco et al., 2021). Nevertheless, obesity is still a risk factor for the occurrence and progression of RCC. According to an epidemiological analysis, about 25% of RCC patients are overweight. Increased BMI is positively related to the prevalence of RCC (Ito et al., 2017; Scelo and Larose, 2018). However, BMI as a diagnostic indicator of obesity still has limitations. For instance, BMI cannot assess the adipose distribution within an individual (Dai et al., 2020). Therefore, whether BMI can be used as a prognostic biomarker for obese RCC patients remains to be further verified. Whether the obesity paradox is applicable to other metabolic diseases has not been widely studied. And whether weight loss in obese RCC patients is the key factor for good prognosis of RCC needs to be clinically confirmed.

## 4 LncRNAs are involved in metabolic regulation of RCC

LncRNAs can interact with miRNAs to affect the activity of miRNAs, thus mediating the occurrence and progression of tumors. MYC, as a carcinogenic gene, is usually activated or up-regulated in cancer. As a member of MYC family, c-MYC can directly manipulate genes related to glucose metabolism, such as hexokinases2 (HK2), glucose transporters (GLUT), phosphofructokinase (PFK) and phosphoglycerate kinase. It has been fully clarified that c-MYC promotes the glycolysis and contributes to tumor progression by up-regulating genes involved in glycolysis pathway and metabolism (Xiao et al., 2017; Gouw et al., 2019). The expression of lncRNA KCNQ1DN was down-regulated in RCC. Gene analysis showed that KCNQ1DN affected glucose intake, inhibited glycolysis pathway, reduced ATP production, and finally hindered biological characteristics of RCC, such as cell proliferation, cell metabolism, migration and invasion. These processes were mediated by suppressing the transcriptional activity of c-MYC and its downstream targets (Yang et al., 2019). LncRNAs can also interact with proteins. Compared with the surrounding non-tumor tissues, lncRNA ROR was highly expressed in RCC tissues. The high expression of ROR would reduce p53 expression, increase c-MYC expression, promote the glycolysis pathway, and further expedite the proliferation of RCC(Shi et al., 2017). Another study manifested that ROR contributed to the stability of c-MYC by combining with heterogeneous nuclear ribonucleoprotein I (hnRNP I) and AU rich RNA binding factor (AUF1) (Huang et al., 2016). It is well known that cancer cells need a lot of energy to maintain their unrestricted proliferation. In response to the energy stress, they will initiate glycolysis. The main transcription factor in glycolysis pathway is c-MYC, while lncRNA FILNC1 can down-regulate c-MYC expression and repress the glycolysis pathway and tumor progression through interaction with AUF1 under energy stress. The lack of FILNC1 in RCC will up-regulate c-MYC, leading to increased glucose uptake and lactic acid production (Xiao et al., 2017). These studies will broaden our understanding of the relationship between lncRNAs and metabolism and cancer.

The most significant characteristics of ccRCC is the transparent morphology formed by adipose accumulation, while lncRNA phospholipid binding protein annexin A3 (AnxA3) is a negative regulator of adipogenic differentiation, with two subtypes of 33kDa and 36 kDa. The expression of 36 kDa AnxA3 was extraordinarily down-regulated in ccRCC cells, whereas 33 kDa AnxA3 was obviously up-regulated, and total AnxA3 was thus dramatically down-regulated (Wu et al., 2013). When adipose culture medium was used to induce ccRCC lipid accumulation, 36 kDa type AnxA3 presented low expression in these cells, indicating that AnxA3 was a negative regulator of ccRCC lipid storage (Bombelli et al., 2020). In consequence, the effect of AnxA3 on RCC and its underlying mechanisms deserve further exploration.

Argininosuccinate Synthase 1 (ASS1) is an enzyme that catalyzes the synthesis of arginosuccinate from aspartic acid and citrulline. Normal cells have the ability to synthesize arginine through ASS1 and argininosuccinate lyase (ASL). However, more than 70% cancer cells have a common metabolic defect, arginine synthesis disorder. Arginine deficiency will mitigate metabolic pathways, including oxidative phosphorylation (OXPHOS), mitochondrial function, glycolysis, etc., (Chen C. L. et al., 2021). It was reported that miR-34a-5p negatively regulated ASS1, while lncRNA 00312 played an anti-tumor role *in vitro* by down-regulating miR-34a-5p and up-regulating ASS1 (Zeng et al., 2020). Zhang et al. found that lncRNA TUG1 (taurine up-regulated gene 1) directly bound to miR-141-3p and negatively regulated miR-141-3p through the ceRNA mechanism, and further activated Wnt/β-catenin signaling pathway. Silence of TUG1 expression could attenuate the up-regulation of 𝛽-catenin, activate c-MYC, accelerate the metabolic pathway of glutamate in RCC, and further promote the proliferation of RCC (Zhang et al., 2020).

**FIGURE 3:**
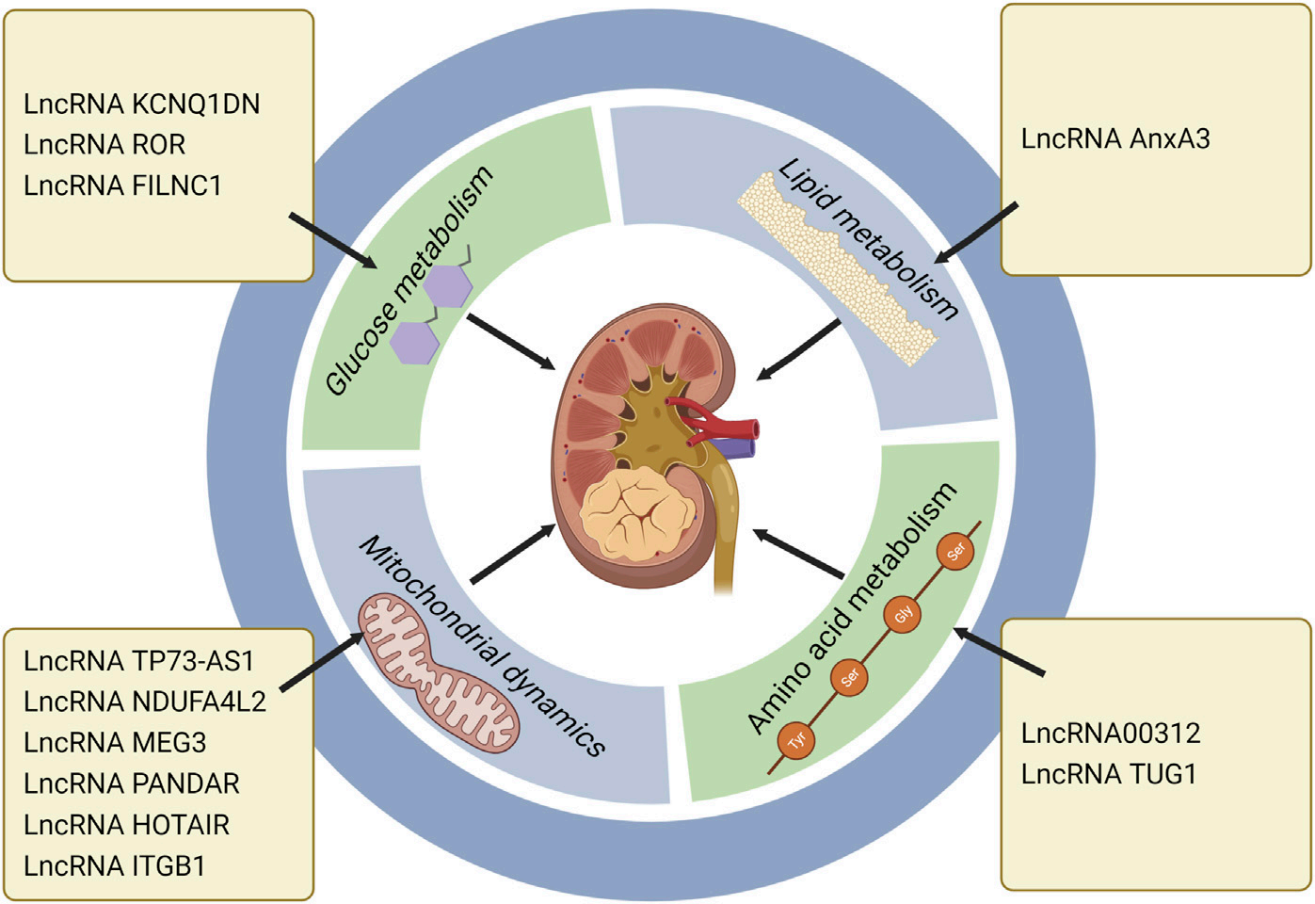
LncRNAs regulate RCC progression through metabolic pathways, especially the roles in glucose metabolism, lipid metabolism, amino acid metabolism and mitochondrial dynamics. LncRNA KCNQ1DN, ROR and FILNC1 affect RCC development *via* regulating glucose metabolism. LncRNA AnxA3 regulates RCC progression through lipid metabolism. LncRNA 00312 and LncRNA TUG1 exert the regulatory roles in RCC through amino acid metabolism. LncRNA TP73-AS1, NDUFA4L2, MEG3, PANDAR, HOTAIR, and ITGB1 influence mitochondrial dynamics and further modulate RCC progression (Created with biorender.com).

Normal mitochondrial function is essential for the metabolic activity of cancer cells. Rapamycin complex 1 (mTORC1) is an important regulator of mitochondrial function, which influences mitochondrial mRNA translation, and renders enhanced mitochondrial ATP production. In addition to energy production, mitochondria also perform other functions, including generation of redox molecules, regulation of cell signal transduction, and production of reactive oxygen species (ROS). Low levels of ROS can promote the proliferation of cancer cells, while excessive ROS will cause cell death (de la Cruz López et al., 2019; Zhang H. et al., 2022). In ccRCC, lncRNA TP73-AS1 accelerated cell proliferation and migration, and mitigated cell apoptosis. Further study of its specific mechanism indicated that knockdown of TP73-AS1 expression could activate the mTOR pathway, and increase ATP production, and thus promote the proliferation of ccRCC. Conversely, TP73-AS1 over-expression inhibited the mTOR pathway, proving that the mTOR pathway was involved in the regulation of TP73-AS1 on ccRCC (Liu et al., 2018). In one study, the transcriptional profile of primary RCC was evaluated, which revealed that the NADH dehydrogenase (Ubiquinone) 1 α subcomplex, 4-like2 (NDUFA4L2) was the highest expressed gene in RCC. NDUFA4L2, a protein encoding HIF-1 target gene, can reduce mitochondrial oxygen consumption and restrict ROS production. Silencing NDUFA4L2 gave rise to declined mitochondrial autophagy, elevated mitochondrial mass, as well as a large number of ROS production (Tello et al., 2011; Lucarelli et al., 2018). RCC is a well-known drug resistant tumor. Over-expression of NDUFA4L2 in RCC can enhance drug resistance. In other words, abnormally expressed NDUFA4L2 will affect the drug resistance of RCC, and NDUFA4L2 may become a new invasive marker of RCC (Lucarelli et al., 2018). Bcl-2 family is a key molecule in the regulation of tumor progression, which is divided into pro-apoptotic members and anti-apoptotic members. As an anti-apoptotic member of the family, Mcl-1 is also a kind of oncogene, which can be regulated at transcriptional, post-transcriptional and post-translational levels (De Blasio et al., 2018). At the post-transcriptional level, PI3K/AKT—mTORC1 can activate Mcl-1 (Gao et al., 2013). Previous studies confirmed that Mcl-1 over-expression was positively correlated with poor survival in breast cancer (Schacter et al., 2014). Mcl-1 inhibited apoptosis by binding Bax and Bak proteins on mitochondrial membrane, thereby releasing cytochrome c (De Blasio et al., 2018). LncRNA MEG3 (maternally expressed gene 3) is a tumor suppressor, which is low expressed in RCC. MEG3 mediates RCC progression by modulating ST3Gal1 transcription and epidermal growth factor receptor (EGFR) sialylation. MEG3 can down-regulate Bcl-2 expression and inhibit cytochrome c release, thus leading to mitochondrial dysfunction and inducing apoptosis of RCC cells (Gong et al., 2020; Mao et al., 2021). LncRNA PANDAR (Promoter of CDKN1 antisense DNA damage active RNA) is significantly up-regulated in tumor tissue. Silencing PANDAR suppressed the expression of Bcl-2 and Mcl-1, further attenuated PI3K/AKT-mTOR signal pathway and facilitated the proliferation of ccRCC(Xu et al., 2017). One study demonstrated that PANDAR might be used as an independent predictor for the overall survival of ccRCC. LncRNA HOTAIR induced dependent death of mitochondrial calcium uptake 1 (MIUC1) in ccRCC by regulating Bcl-2 and cytochrome c to affect mitochondrial membrane potential (Henzi and Schwaller, 2015; Ding et al., 2018; Zhang H. et al., 2022). The expression of lncRNA ITGB1 in ccRCC is obviously higher than that in adjacent normal tissue. Over-expression of ITGB1 in ccRCC strikingly reduced Mcl-1 expression, indicating that ITGB1 promotes cancer progression by modulating Mcl-1 (Zheng et al., 2019) (Table 2) (Figure 3).

**TABLE 2:** RCC biological behavior regulated by lncRNAs *via* metabolic pathways.

LncRNA	Metabolic pathways	Mechanisms	Expression of RCC	Biological behavior of RCC	References
LncRNA KCNQ1DN	glucose metabolism	c-MYC	down-regulated	apoptosis	Yang et al. (2019)
LncRNA ROR	glucose metabolism	p53/c-MYC、hnRNP Ⅰ/AUF1/c-MYC	up-regulated	proliferation	Shi et al. (2017), Huang et al. (2016)
LncRNA FILNC1	glucose metabolism	AUF1/c-MYC	down-regulated	apoptosis	Xiao et al. (2017)
LncRNA AnxA3	lipid metabolism	\	down-regulated	apoptosis	Wu et al. (2013), Bombelli et al. (2020)
LncRNA 00312	amino acid metabolism	miR-34a/5p-ASS1	down-regulated	apoptosis	Zeng et al. (2020)
LncRNA TUG1	amino acid metabolism	miR-141-3p/Wnt/β-catenin/c-MYC	down-regulated	apoptosis	Zhang et al. (2020)
LncRNA TP73-AS1	mitochondrial dynamics	mTOR	up-regulated	proliferation	Liu et al. (2018)
LncRNA NDUFA4L2	mitochondrial dynamics	HIF-1	up-regulated	proliferation	Lucarelli et al. (2018)
					Tello et al. (2011)
LncRNA MEG3	mitochondrial dynamics	ST3Gal1/EGFR/Bcl-2/cytochrome c	down-regulated	apoptosis	Mao et al. (2021), Gong et al. (2020)
LncRNA PANDAR	mitochondrial dynamics	Bcl-2/Mcl-1/PI3K-AKT- mTOR	up-regulated	proliferation	Xu et al. (2017)
LncRNA HOTAIR	mitochondrial dynamics	MIUC1/Bcl-2/cytochrome c	up-regulated	proliferation	Ding et al. (2018), Henzi and Schwaller (2015)
LncRNA ITGB1	mitochondrial dynamics	Mcl-1	up-regulated	proliferation	Zheng et al. (2019)

## 5 Clinical applications of lncRNAs *via* targeting metabolism in RCC therapy

In recent years, with the emergence of novel drugs such as immune checkpoint inhibitors, human anti-tumor therapy has reached a new level. However, the therapeutic efficacy is limited, mainly because most patients are not sensitive to immune checkpoint inhibitors, and the mechanism is indefinite. Accordingly, anti-tumor therapy *via* targeting metabolism may be a new research direction (Syn et al., 2017).

It was reported that FILNC1 (FoxO-induced long non-coding RNA 1) knockout promoted RCC progression by up-regulating c-MYC to enhance glycolysis level. Due to high expression of FILNC1 in RCC, whether it can be used as a biomarker for clinical RCC diagnosis needs further exploration (Xiao et al., 2017). LncRNA00312 was low expressed in RCC, and inhibited the proliferation and invasion of cancer cells *in vitro* by elevating ASS1. Clinical studies show that the patients with low-expressed LncRNA00312 had poor prognosis (Zeng et al., 2020). It is well known that everolimus and temsirolimos are in clinical development, which can block the mammalian target of rapamycin (mTOR) signal pathway and in turn enhance the expression of HIF(Hudes et al., 2007; Motzer et al., 2008). However, these drugs only act on a few downstream target genes of HIF. Whether better anti-cancer effect can be achieved by interrupting HIF1D6FC; transcription activity to regulate all genes, is currently being evaluated.

At present, the therapeutic options for advanced metastatic RCC are limited, mainly using receptor tyrosine kinase inhibitors (RTKIs), such as sunetinib and sorafenib, but most patients develop drug resistance after half a year (Motzer et al., 2007; Bergers and Hanahan, 2008; Molina et al., 2014). One study showed that lncRNA SNHG12 was significantly up-regulated in sunetinib-resistant RCC, which was associated with poor clinical outcomes. This result indicates that knocking down SNHG12 might provide a new therapeutic target for reversing the resistance to sunitinib (Liu et al., 2020). The most effective treatment for end-stage renal diseases is kidney transplantation (KT) nowadays, in which post-transplant diabetes mellitus (PTDM) is the most common factor affecting the clinical effect of KT. Since hyperglycemia itself is a risk factor for PTDM, blood glucose should be strictly controlled before and after transplantation. The regulatory effect of LncRNAs on glucose metabolism can not only alter the incidence of PTDM, but also influence the prognosis of patients (Chang and Wang, 2019; Li et al., 2019). In the future study, it was proposed that more attention be paid to the hypoglycemic effect and potential targets of lncRNAs in the clinical therapies.

## 6 Conclusion and future prospects

The published data demonstrate that lncRNAs present different expression in RCC. RCC progression can be manipulated by lncRNA over-expression or knockdown. Up to now, the regulatory effect of lncRNAs on RCC has been proved by a large number of studies. And the research on lncRNA targets and signal pathways is also relatively extensive, such as PI3K/AKT, mir4429-UBE2C, miR-34c-5p/MMP2, miR34/miR449/STAT3 and UPF1-Wnt/β-catenin. This review mainly summaries the regulatory roles of lncRNAs as ceRNAs by binding to miRNAs. It is worth mentioning that the mechanisms of lncRNAs on protein coding genes related to tumorigenesis is not confined to miRNAs mediated ceRNA model. LncRNAs can also be directly combined with mRNAs or proteins to affect their expression and stability. More studies are expected on the mechanisms of lncRNAs to be carried out, which will broaden the new approaches for lncRNAs to regulate tumor progression. However, there are relatively few studies on RCC progression mediated by lncRNAs through metabolic pathways. As a kind of novel marker for malignant tumors, lncRNAs can modulate metabolism-related genes (c-MYC, ASS1, mTORC1), and further exert complex impacts on tumor progression, which expands our understanding of cancer metabolism. Currently, since the metabolic regulation of lncRNAs on tumors is not completely clarified, the relevant clinical treatment is limited, which needs further exploration. Furthermore, some controversial phenomena mentioned in this review, including the obesity paradox and the targeting effect of SCD1 inhibitors, have not been illuminated with reliable data. In the past 50 years, the detection and clinical evaluation modes of RCC have made great progress, and the pathological diagnosis and clinical guidance scheme of RCC are also becoming more and more reliable. Non-etheless, advanced RCC is still incurable, and life span can only be prolonged by treatment. Consequently, there is an urgent need to search for appropriate lncRNAs as biomarkers of RCC and further improve the clinical therapy for RCC *via* lncRNA manipulation. Our understanding on how lncRNAs influence RCC progression *via* metabolism regulation is only the tip of the iceberg, and there are still many questions worth further exploration.
